# A Novel Cooperative Opportunistic Routing Scheme for Underwater Sensor Networks

**DOI:** 10.3390/s16030297

**Published:** 2016-02-26

**Authors:** Seyed Mohammad Ghoreyshi, Alireza Shahrabi, Tuleen Boutaleb

**Affiliations:** School of Engineering and Built Environment, Glasgow Caledonian University, Lanarkshire, Glasgow G4 0BA, UK; A.Shahrabi@gcu.ac.uk (A.S.); T.Boutaleb@gcu.ac.uk (T.B.)

**Keywords:** routing protocol, void area, underwater sensor network

## Abstract

Increasing attention has recently been devoted to underwater sensor networks (UWSNs) because of their capabilities in the ocean monitoring and resource discovery. UWSNs are faced with different challenges, the most notable of which is perhaps how to efficiently deliver packets taking into account all of the constraints of the available acoustic communication channel. The opportunistic routing provides a reliable solution with the aid of intermediate nodes’ collaboration to relay a packet toward the destination. In this paper, we propose a new routing protocol, called opportunistic void avoidance routing (OVAR), to address the void problem and also the energy-reliability trade-off in the forwarding set selection. OVAR takes advantage of distributed beaconing, constructs the adjacency graph at each hop and selects a forwarding set that holds the best trade-off between reliability and energy efficiency. The unique features of OVAR in selecting the candidate nodes in the vicinity of each other leads to the resolution of the hidden node problem. OVAR is also able to select the forwarding set in any direction from the sender, which increases its flexibility to bypass any kind of void area with the minimum deviation from the optimal path. The results of our extensive simulation study show that OVAR outperforms other protocols in terms of the packet delivery ratio, energy consumption, end-to-end delay, hop count and traversed distance.

## 1. Introduction

**Figure 1:**
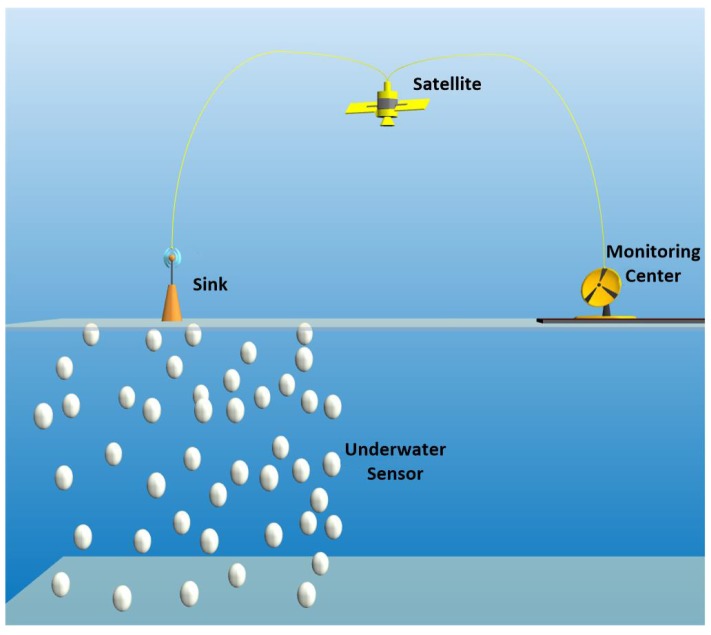
Underwater sensor network.

Nowadays, resource discovery in the underwater environment has become one of the important goals to reduce dependency on land resources. However, it is a difficult and costly task to monitor and discover the underwater environment. Underwater sensor networks (UWSNs) have recently attracted much attention due to their significant ability in ocean monitoring and resource discovery. Due to restrictions on the use of radio waves, acoustic transmission is most commonly used in the underwater environment. Required data are collected by the underwater sensors and directed towards the sink on the surface. Afterwards, the sink can transmit collected information to the monitoring centre via satellite for further analysis [1,2,3,4], as shown in Figure 1.

Some unique features of UWSNs make data forwarding in this environment a challenging task. This includes node movement, low available bandwidth, slow propagation speed, high deployment cost and a lossy environment [5,6,7]. It also should be mentioned that the Global Positioning System (GPS) cannot be used in an underwater environment as a localisation system because of the quick attenuation of its waves in water [5]. Furthermore, nodes cannot be aware of their positions by pre-configuration, because they are not stationary due to the water current. Nevertheless, the depth of each node in the water can be estimated through an embedded pressure gauge [8]. Then, depth information can be used during the data forwarding procedure.

The presence of void areas, a high bit error rate and energy conservation are perhaps the most challenging issues from the perspective of routing protocols in UWSNs. A void communication area is a three-dimensional region between underwater nodes that lacks any nodes inside (similar to holes). The void area can prevent communication between some of the nodes in the network [9]. There are various reasons for the presence of void areas, such as sparse topology, temporary obstacles, unreliable nodes or links, *etc.* [10]. In most cases, the lack of employing enough sensor nodes, due to their high cost, while covering a large monitoring area might lead to sparse deployment of the sensors and, consequently, the creation of some void area. Moreover, the relocation of underwater sensor nodes by the water current can potentially create a void area [11,12].

On the other hand, the adverse characteristics of the underwater channel can cause a high bit error, resulting from high attenuation, channel fading, noise, Doppler spread, *etc.* The communication channel quality varies at different ocean depths under varying pressure, temperature and salinity. The limited bandwidth of acoustic transmission also reduces the efficiency of communication between underwater nodes [13,14]. Generally, nodes are considered connected to each other if the transferred signal between them can be decoded without any error.

In terms of energy consumption, there are also some restrictions due to the difficulties of replacing or recharging batteries, which are the main energy supply for the nodes, in the adverse and often deep underwater environment. In addition, underwater sensors consume more energy than terrestrial sensors because they use acoustic communication [15,16,17]. Thus, employing an efficient routing protocol is quite essential to prolong the whole network lifetime.

Opportunistic routing is a promising scheme in sensor networks because of its remarkable ability to increase transmission reliability and network throughput. In this way, packet forwarding is enhanced by taking advantage of simultaneous packet reception of neighbouring nodes of a forwarding node and their collaboration to forward the packet [18,19,20]. However, applying a terrestrial opportunistic routing protocol in UWSNs without considering its specific features is not possible in most cases. In the underwater environment, forwarding set selection without a hidden terminal and prioritizing them are affected by features like a high error bit rate, energy consumption, node movement and slow propagation speed. Furthermore, some terrestrial opportunistic protocols are GPS-based, which make them inappropriate for the GPS-denied underwater environment.

The redundant packet transmission issue is one of the influential factors on the opportunistic routing performance. When a group of candidate nodes are selected to collaboratively forward a packet while placed out range of each other, they cannot notice the transmission of any packet by other candidates. Thus, each forwarding node sets its forwarding timer and forwards the packet separately, resulting in more collisions and energy consumption. If the forwarding nodes are selected within the transmission range of each other (without any hidden node), this increases the chance of hearing the packet transmission by other higher priority candidate nodes, although there is no absolute guarantee, because of other factors, like shadow zone occurrence [21]. Nevertheless, some underwater routing protocols (e.g., Adaptive Hop-by-Hop Vector-Based Forwarding (AHH-VBF) [22], HydroCast [14], Void-Aware Pressure Routing (VAPR) [8]) take advantage of a group of forwarding nodes in the vicinity of each other with a timer-based coordination to eliminate the duplicated packet problem in the routing layer. It should be noticed that the hidden terminal problem still may exist in the other layers of the network, which is out of the scope of this work.

In this paper, we propose a new opportunistic void avoidance routing (OVAR) protocol in order to increase the throughput and reliability in the sparse and lossy underwater environment while imposing less overhead in comparison to those protocols using high cost localisation to obtain their geographic coordinates in this environment. Furthermore, unlike the stateful protocols, which require global topology information, OVAR only depends on the information provided by one-hop neighbouring nodes. Each forwarding node selects its forwarding set with the aid of information obtained from the distributed beaconing mechanism initiated from the sink node. OVAR is able to bypass void areas before being stuck in a void node and simultaneously selects a group of candidate nodes with the highest advancement towards the sink. The forwarding set is selected in such a way that its members can hear each other and suppress duplicate transmissions, which leads to a decrease in energy consumption and congestion. In order to prevent energy wasting in a high-density forwarding set, the number of receiving nodes can be appropriately adjusted.

The rest of this paper is organised as follows: In Section 2, we review the related work in this field. In Section 3, the details of the OVAR protocol are presented after introducing the network architecture and void problem. In Section 4, we make theoretical analyses about the energy consumption and reliability. Section 5 evaluates the performance of OVAR through simulations. In Section 6, we conclude the paper and discuss future work.

## 2. Related Work

In this section, we review some geographic routing protocols in the UWSN and how they take advantage of the opportunistic data forwarding to deal with the void and channel fading. It was mentioned that GPS does not work in the underwater environment; however, some studies still assume that underwater nodes can obtain their 3D geographic coordinates with the aid of the localisation service [23,24,25], which is reported to be a challenging issue in the underwater environment [26].

Some routing protocols, such as Vector-Based Forwarding (VBF), Hop-by-Hop Vector-Based Forwarding (HH-VBF) and Adaptive Hop-by-Hop Vector-Based Forwarding (AHH-VBF) [22,27,28] are location-based greedy routing in which forwarding nodes are selected within a virtual pipeline facing toward the destination. These protocols are receiver-based, which means that when a forwarding node broadcasts a packet, candidate nodes decide whether to collaborate in the packet forwarding [29]. These protocols also consider a desirableness factor to select forwarding candidates among the nodes inside the pipe. In order to reduce the latency, the nodes are selected in a way that a packet is forwarded using the longest possible hop from the transmitter while maintaining its closeness to the routing vector. However, in the underwater environment, the likelihood of bit error increases with increasing the traversed distance. They try to compensate this defect by increasing the radius of the pipe and involving more forwarding nodes, which, however, causes a higher probability of collisions and, hence, wastes energy. In addition, increasing the radius of the pipe does not help to resolve the problem of the void area, which mostly occurs in sparse networks. The transmitter cannot utilise the nodes outside the pipe to bypass a void area located in the pipe. Moreover, these protocols suffer from hidden terminal nodes, because the neighbouring nodes of the sender can be out of range of each other (e.g., be placed in different directions of the pipe).

Vector-Based Void Avoidance (VBVA) [11] is proposed to mitigate the negative impact of void communications on the vector-based routing protocols, such as VBF and HH-VBF [27,28]. VBVA exploits two approaches, vector-shift and back-pressure, for dealing with the convex and concave voids, respectively. However, the recovery procedure of VBVA is too complicated to be performed in the real underwater environment. VBVA lets the packets be trapped in a concave hole, and then, it tries to recover them with a time-consuming procedure, leading to higher end-to-end delay.

Relative Distance Based Forwarding (RDBF) [16] is another routing protocol that similarly relies on the use of location-based coordinates. In this protocol, packets are relayed through the nodes with the nearest geographical distance to the sink node. RDBF does not limit the forwarding nodes in a pipe or other geometric shape; however, it considers a fitness factor as a threshold to limit the number of forwarding nodes. RDBF also suffers from the high bit error rate, because it mostly relies on the nodes with the shortest path to the sink. In addition, RDBF does not represent a recovery mode to deal with the packets that are stuck in a local maxima node.

A group of routing protocols, like GEographic and opportunistic routing with Depth Adjustment-based topology control for communication Recovery (GEDAR) [30], Depth-Controlled Routing (DCR) [31] and Greedy Routing with Distributed Topology Control (GR+DTC) [12], exploits a network topology control scheme, which enables them to deal with the communication void problem. In this way, all void nodes can move vertically to be connected to a non-void node. Network topology control improves the network connectivity and diminishes the impact of the void by utilising the vertical movement capability of the nodes. However, this technique consumes high energy for topology adjustment, which only can be justified in the long-term and non-time-critical applications.

In another group of studies, depth information is employed to route the packets towards their destinations (one of the sonobuoys on the water surface). Depth-Based Routing (DBR) [26] is the first depth-based routing protocol proposed for UWSNs. Nevertheless, forwarding set selection is not performed in an optimal way (having the duplicated packets problem), and neither proposes any recovery method to solve the void problem. Depth-Based Multi-hop Routing (DBMR) and Energy-Efficient Depth-Based Routing (EEDBR) [32,33] are also proposed in this category, which consider the residual energy of the nodes in their forwarding set selection; however, they still provide no solution for the local maxima nodes.

On the other hand, HydroCast [14] and Void-Aware Pressure Routing (VAPR) [8] represent the pressure-based routings, which are enhanced by opportunistic data forwarding and void handling. These protocols are sender-based approaches, which means that each forwarding node selects the candidate nodes and puts their IDs in the packet header. The receiving node collaborates in packet forwarding if its ID is included in the packet header [29]. These protocols try to select a subset of forwarding candidates with maximum advancement towards the destination, while also addressing the hidden terminal problem. However, HydroCast relies on the use of a 2D surface flooding method to discover a recovery path for local maxima nodes in the near-surface layer. More importantly, void areas can appear in deeper regions of the water, which is not considered in this protocol.

VAPR tries to bypass void areas by holding information of up to two-hop neighbouring nodes, which imposes high overhead to the system. Moreover, the beaconing procedure in VAPR (for a multi-sink architecture) is not properly utilised in a way that beacons carry additional useful information in addition to the hop count. For this reason, in VAPR, each node is forced to periodically measure the distance to its neighbouring nodes and to broadcast the measured information for them. As another problem, the packet can only be forwarded up or down depending on the selected direction, which cannot utilise subsets of nodes in the horizontal direction (including nodes with a lower depth and a higher depth together in the forwarding set). Subsequently, in facing a convex void, packets will traverse a longer distance because they cannot be forwarded in a horizontal direction to bypass the void. Furthermore, this leads to less available candidates and, hence, increases the packet failure probability.

## 3. Opportunistic Void-Avoidance Routing Protocol

In this section, we present our OVAR protocol in detail.

### 3.1. Problem Description

During the packet forwarding, if a relay node cannot find any qualified node with a positive progress towards the destination, the packet may be dropped, even though there exists a topologically-valid path from the sender to the destination. This phenomenon is called the local maxima or void problem. The performance of greedy-based routing protocols significantly degrades in the presence of a void area. The characteristics of an underwater sensor network can make the problem even more challenging. The mobility of most underwater nodes and three-dimensional holes in the routing path can lead to more packet failures [11]. Moreover, some moving objects, like a ship, can temporarily create a void area by blocking the communication between two parts of the network [22]. In dense networks, void areas can arise temporarily and with small volumes in some regions. On the contrary, sparse networks include many void areas, which severely affect routing performance. Some existing protocols often ignore void handling in their routings or employ high overhead methods to mitigate its effect.

On the other hand, the underwater environment has higher path loss and more ambient noises in comparison to the terrestrial physical layer. The main sources of the noise include turbulence, shipping, waves and thermal noise [13]. Moreover, packet loss depends on the traversed distance and the transmission power of the underwater acoustic signal [14,34]. Thus, packet forwarding is more likely to be successful if packets are relayed over multiple short distances instead of traversing over long distances. These factors can influence the design of underwater sensor protocols, which are not properly resolved in the majority of the proposed protocols. Applying some simplistic methods, such as increasing the number of forwarding nodes or increasing the transmission power, mostly lead to a waste of energy overall.

Energy consumption is another major concern in UWSNs, because it is hard to replace or recharge the sensor batteries in the harsh underwater environment. In the underwater acoustic networks, the energy consumed by the sensors is much more than what is consumed by the regular sensors in the terrestrial networks [35]. Therefore, energy efficiency is an essential requirement of routing protocols in UWSNs. To this end, it should be noted that the energy consumed by data processing is significantly less than that of data transmission [36,37].

### 3.2. System Model

In contrast to the terrestrial networks in which the network topology is simplified into a 2D one, an underwater sensor network has a 3D network topology. In our underwater acoustic sensor network model, a single sink is considered on the water surface, which is equipped with an acoustic modem for underwater communication and a radio modem for out of water communication with the monitoring centre [27,28]. Anchored nodes are located at the bottom of the ocean in the predetermined locations to collect the information and deliver it to the sink by using the relay nodes, which are located at different levels in between [22,27,28]. Relay nodes and anchored nodes use acoustic signals to transmit the packets. Packets can be forwarded at longer distances by using the higher intensity of acoustic pressure. Moreover, the velocity of an acoustic signal depends on the varying pressures and temperatures [21].

We assume that each node knows its current depth (*i.e.*, vertical distance from each node to the water surface) by using an embedded depth sensor [26]. Moreover, nodes can obtain their hop count distance to the sink with the aid of distributed beaconing [8]. Nodes randomly move in the horizontal direction because of the water current, and their small vertical movements are negligible. The batteries are the energy suppliers of the underwater sensor nodes. Nodes are homogeneous in terms of energy consumption and transmission range. The Thorp model is used for designing the underwater acoustic propagation and adjusting the transmission power [22,28]. Moreover, we consider a lossy channel in which path loss and bit error depend on the traversed distance and signal frequency. The path loss or attenuation over distance *d* with the signal frequency *f* is defined as follows [13]:

(1) $$A\left(d,f\right)=A_{0}d^{k}\alpha {\left(f\right)}^{d}$$

where $A_{0}$ represents a unit-normalizing constant and *k* is the geometric spreading factor, which is set to 1.5 for practical scenarios. Furthermore, the absorption coefficient α(f) is defined by the Thorp formula. The ratio of the signal power, which contains meaningful data, to the unwanted signal power (*i.e.*, noise) is defined as the signal-to-noise ratio (SNR). By considering the attenuation formula, the signal-to-noise ratio over distance *d* with the signal frequency *f* can be expressed as follows [13]:

(2) $$S\;N\;R\left(d,f\right)=\frac{P\;R(f)}{A(d,f)P\;N(f)}$$

where PR(f) and PN(f) indicate the transmission power of the forwarding node with frequency *f* and the underwater environment noise, respectively. In order to decode the received signal without error, SNR at the receiver should be higher than a detection threshold. The ambient noise in the underwater environment includes four main components of turbulence $P\;N_{t}\left(f\right)$, shipping $P\;N_{s}\left(f\right)$, waves $P\;N_{w}\left(f\right)$ and thermal energy $P\;N_{th}\left(f\right)$, which can be expressed as [22]:

(3) $$P\;N\left(f\right)=P\;N_{t}\left(f\right)+P\;N_{s}\left(f\right)+P\;N_{w}\left(f\right)+P\;N_{th}\left(f\right)$$

These noises are dominant in the different frequency regions, which can affect the communication channel throughput.

### 3.3. OVAR Overview

In order to properly address the void problem and also to deal with the lossy nature of the underwater acoustic channel, OVAR uses an opportunistic routing algorithm to increase the transmission reliability and also the network throughput while excluding all routes leading to a void area. By taking advantage of the broadcast nature of the acoustic signal, forwarding nodes locally collaborate on packet forwarding with very low overhead.

Having a single permanent destination, in the single-sink model, or a number of destinations, in the multi-sink model, is a unique useful feature in developing void-aware routing protocols for UWSNs, which has been perhaps neglected in most routing protocol developments in this field. Using this feature, the process of establishing a void avoidance route for all of the nodes in the network to their destination(s) can be initiated by the sink(s) and cascaded down by intermediate nodes, similar to the route establishment phase of some distance vector routing protocols in wireless *ad hoc* networks. In order to obtain reachability information and neighbouring nodes’ discovery, each node periodically broadcasts a beacon, which includes the hop count information (proximity of nodes to the sink) and also some neighbouring information for updating the neighbouring tables. The beaconing mechanism has already been implemented and utilised by some MAC protocols [38,39] for neighbouring nodes’ discovery. This mechanism can be augmented to support the hop count information required by OVAR without imposing new overhead.

It should be noted that OVAR is a soft-state routing protocol. In a soft-state routing protocol, some reachability information (e.g., hop count distance, forwarding direction) can be provided and kept in each node [8]. However, the scalability of the routing protocol should not be sacrificed. Therefore, there is a trade-off between the protocol’s scalability and reachability information at each node. Although this information gives a general view of each node, all routing decisions should be made locally to hold the scalability of the protocol. Moreover, regarding the dynamicity of underwater currents (slow nodes’ movement), it is assumed that routing can be accomplished much faster than topology changes. Therefore, the cost of information distribution is negligible against the cost of the routing and packet recovery. Therefore, no routing path is maintained in each node in OVAR apart from some reachability information, which is useful for efficiency, but not essential, as it can be regenerated or updated if needed [8].

OVAR employs a hop-by-hop forwarding set selection to deliver packets to the sink. Each packet holder uses local information of hop distance and packet advancement to determine its own forwarding set. In addition, the forwarding set should prevent the hidden terminal problem, which is caused by including the nodes that are out of range of each other. In order to manage the energy, the number of collaborative nodes can be adjusted according to the density of the network. Afterwards, in order to prioritise the multiple forwarding nodes, each node considers its depth as the second metric to set a relaying timer. The node with the highest priority (lowest depth) transmits the packet earlier, and other low priority nodes can drop the packet after hearing the transmission. This suppression mechanism along with the selecting of a path with a lower hop count leads to more energy savings and a higher delivery ratio. By employing hop-by-hop forwarding set selection, OVAR is highly scalable to be used in large underwater sensor networks. Finally, OVAR automatically excludes all of the routes leading to void areas and, therefore, does not need to switch any high overhead recovery mode for void bypassing.

### 3.4. Beaconing Model

We consider *S* as a single sink on the surface for collecting the information. Other nodes, including relay nodes and anchored nodes, can be shown by $V=\{R_{1},R_{2},...,R_{m}\}$. Let m=|V| denote the number of nodes in *V*. We define $N(R_{i})$ as the set of $R_{i}$ neighbouring nodes. Based on $R_{i}$ members’ hop count values, $N(R_{i})$ can be partitioned as follows:

(4) $$N\left(R_{i}\right)=L\left(R_{i}\right)⋃E\left(R_{i}\right)⋃H\left(R_{i}\right)$$

where $L(R_{i})$, $E(R_{i})$ and $H(R_{i})$ indicate disjoint neighbouring sets of $R_{i}$ with lower, equal and higher hop count values, respectively. Each node in *V* locally holds a table about its neighbouring nodes and classifies them based on the partitioning criteria expressed in Equation (4).

At the beginning of the beaconing process, all of the nodes in *V* are isolated from each other, and their hop count value is set to a maximum value, to show no connectivity with *S*. Node *S* is the final destination on the surface, and accordingly, its hop count number is set to zero. In our beaconing model, node *S* along with all of the nodes in *V* periodically propagate a beacon, including their ID, depth, hop count value and all neighbouring nodes in subset $E(R_{i})$ (neighbouring nodes with the same hop count value as the sender). Nodes with the maximum hop count value are exempted from the beaconing until they find a path to the sink. The sink node initiates the beaconing process and gradually is cascaded down to the network. The beacon interval for each node is considered as $T_{update}$.

In order to facilitate a quick convergence in the network, a node resets its beacon timer and propagates a new beacon, or triggered beacon, as soon as its hop count value is changed. A triggered beacon can subsequently initiate some new triggered beacons, gradually leading to the synchronisation of beacons. This is an issue due to periodic beacon propagation. Synchronised beacons can collide, due to long propagation delay and also the existence of hidden nodes, and cause delays and wasting of bandwidth. The beacons are not initially synchronised, but the timers across the network become globally synchronised over time. To prevent this issue, a random amount of time is added to the beacon interval at each node. This random time ranges from 0% to 20% of the determined beacon interval, $T_{update}$. In this way, the beacon interval varies randomly in a range from $T_{update}$ to $\left(T_{update}+T_{update}\times 20\%\right)$.

Depending on the hop count value of the beacon, the receiving node decides how to deal with it. Upon receiving a beacon with a lower hop count, the receiving node updates its hop count value, holds the sender’s ID in its subset *L* and attaches its depth, as well as all other existing IDs within the beacon to the sender entry in the table. If a node receives other beacons with the same lower value, it will also add them to the table in the same manner. Since all nodes periodically broadcast a beacon, the receiver knows all of its neighbours with a lower hop count (all next available nodes to relay the packets during the packet forwarding stage). On the other hand, when a node receives a beacon with the same hop count value as its own value, it only holds the sender’s ID in its subset *E* and broadcasts it with the next beacon. Furthermore, the receiving node drops all of the beacons with the higher hop count value.

According to the information extracted from the table, each node can form its own adjacency graph. Only nodes in the subset *L* are considered to be included in the graph, and other nodes, which are inaccessible by the sender, can be removed from the table. It can simply be realized by checking the beacons from which neighbours directly received by the node.

Upon changing the hop count value in each node (e.g., finding a shorter path), the table is updated based on the current available path, and it sends out a beacon with a new hop count value and also resets the beacon timer. Moreover, nodes employ $T_{invalid}$, which shows how long a path is valid at each node. If a node cannot sense any neighbouring node with a lower hop count in its vicinity in this time interval, it should determine a new hop count value based on the recently received, and still valid, beacons. The routing performance depends on the assigned value for $T_{update}$ in a way that a higher value leads to the invalidity of the vicinity information and a lower value imposes high communication overhead. According to the mobility pattern and speed of underwater nodes, $T_{update}$ should be carefully determined.

### 3.5. Routing Algorithm

In the OVAR routing algorithm, we select a forwarding set based on two metrics: packet delivery probability and packet advancement. In this section, we first explain how the packet delivery probability can be estimated from receiving beacons. We then specify how packet advancement is modelled in our routing algorithm.

**Figure 2:**
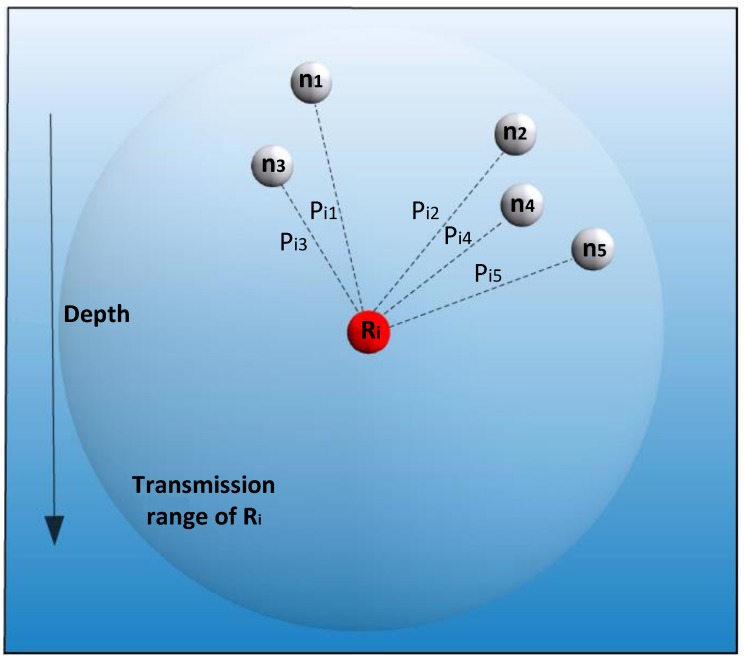
Example in which node $R_{i}$ is forwarding a packet.

The OVAR routing algorithm is divided into three phases. First, an adjacency graph is constructed at every node, and using a heuristic, a clique sub-graph with the maximum expected packet advancement (EPA) is created to ensure that hidden nodes are removed from the forwarding set and the chance of successful delivery is increased. Second, the number of forwarding nodes in the forwarding set is adjusted to make a trade-off between reliability and energy consumption. Finally, the holding time is calculated at each candidate node before forwarding the packet. In order to illustrate the protocol, we consider a local OVAR scenario like the one presented in Figure 2.

#### 3.5.1. Relationship between Packet Delivery Probability and Transmission Distance

Assume node $R_{i}$ intends to send a packet to the sink *S*, and $L\left(R_{i}\right)=\left\{n_{1},n_{2},\ldots ,n_{c}\right\}$ shows the available candidates of node $R_{i}$ (neighbouring nodes with lower hop count values), which are ordered increasingly based on their depth values. Let $c=|L(R_{i})|$ denote the number of candidates in $L(R_{i})$. Node $R_{i}$ is aware of the packet delivery probability of its neighbours. For instance, if $R_{i}$ has received a beacon from $n_{k}$, 1≤k≤c, can calculate pairwise distance $Dist(R_{i},n_{k})$ based on the receiving signal power from the beacon or by using the time of arrival (ToA) [23]. In this way, node $R_{i}$ can calculate all pairwise distances between itself and its neighbouring nodes and add them to its neighbouring table. Thus, all nodes in $L(R_{i})$ can be associated with a packet delivery probability $P_{ik}$ (1≤k≤c), which can be calculated, as explained later in this section, based on the distance from node $R_{i}$ to $n_{k}$. Node $n_{k}$ is a neighbouring node of $R_{i}$ when $P_{ik}>P_{T}$, where $P_{T}$ represents a probability threshold. Otherwise, the sent packet cannot be decoded free of error. Moreover, we assume that the packet delivery probability on each acoustic link is independent.

According to the model used in [14], the bit error probability over distance *d* can be calculated as follows:

(5) $$P_{e}\left(d\right)=\frac{1}{2}\left(1-\sqrt{\frac{S\;N\;R_{avg}\left(d,f\right)}{1+S\;N\;R_{avg}\left(d,f\right)}}\right)$$

where $S\;N\;R_{avg}\left(d,f\right)$ is the average signal-to-noise ratio over distance *d*. The bit error probability increases by increasing the distance due to channel fading. Moreover, a packet (from node *i* to node *j*) with size *n* bits can be delivered over distance *d* with the probability $P_{ij}$ [14]:

(6) $$P_{ij}={\left(1-P_{e}\left(d\right)\right)}^{n}$$

Let *F* denotes $R_{i}$’s forwarding set, including all of the nodes used in the opportunistic data forwarding. Let r=|F| denote the number of nodes in *F*. Now, our first goal is to select the subset *F* from $L(R_{i})$ in a way that it can maximize the packet delivery probability and resolve the hidden terminal problem in the lossy underwater environment.

Obviously, a packet transmission obtains more of a chance of delivery if more forwarding nodes are involved in the packet forwarding. With r=1, only one node from $L(R_{i})$ is selected for packet forwarding, and therefore, the successful delivery chance is limited to the packet delivery probability of a single node. For instance, in Figure 2, if we just select node $n_{1}$, the delivery probability is equal to $P_{i1}$. A traditional routing protocol without opportunistic routing might ideally achieve $max(P_{i1},P_{i2},...,P_{ic})$ packet delivery in each step towards the destination, which is not suitable for the lossy underwater acoustic channel. On the other hand, by maximizing the forwarding set size, *i.e.*, r=c, all neighbouring nodes with a lower hop count take part in the packet delivery. Although this certainly increases the chance of packet delivery, it also increases the energy consumption and also the network congestion. Moreover, involving nodes without considering the hidden terminal problem may result in redundant paths and packet collisions. The three-dimensionality of the underwater environment makes the hidden terminal problem even worse due to the existence of some neighbouring nodes in different directions.

#### 3.5.2. Packet Advancement

To specify the priority of relaying nodes, we define a fitness factor, *α*, which represents the depth difference between sender’s depth, $D_{s}$, and receiver’s depth, $D_{r}$, in a normalised value as follows:

(7) $$\alpha =\frac{D_{s}-D_{r}}{R}\;\left(-1\leq \alpha \leq 1\right)$$

where *R* is the transmission range of sensor nodes. According to the fitness factor, a relay node with a lower depth has higher priority to relay the packets as it is closer to the surface where the sink is located. The negative value of the fitness factor indicates that the receiver node is located below the sender, perhaps due to the presence of a void area in the routing path. In contrast to the majority of greedy routing protocols, OVAR gives these kinds of nodes (with a higher depth than the sender and maybe having a higher geographical distance to the sink) the chance to participate in the packet forwarding to bypass the void areas. However, these nodes still can be prioritised based on the lower depth due to the fact that the packet most likely should be relayed upward over the next step to become closer to the final destination on the surface. In order to use this value in our calculations, we further normalise this value to be placed in the range [0,1] as follows:

(8) $$\beta =\frac{1}{2}\left(\alpha +1\right)\;\left(0\leq \beta \leq 1\right)$$

Now, we can explain three phases of our routing algorithm: forming the adjacency graph and selecting the best forwarding set, adjusting the number of forwarding nodes in the forwarding set and, finally, holding time calculation. Algorithm 1 details the two phases of OVAR: forwarding set selection and adjusting the number of forwarding nodes.

**Algorithm 1** OVAR routing algorithm.
1:**procedure** ForwardPacket($R_{i}$, P)   2:    **if** *forwarding timer expired* **then**   3:        $F(R_{i})=\emptyset$   4:        $L\left(R_{i}\right)=\left\{n_{k}|1\leq k\leq c\right\}$  5:        $G\left(L\left(R_{i}\right)\right)=NeighGraph\left(L\left(R_{i}\right),Table\left(R_{i}\right)\right)$  6:        $F\left(R_{i}\right)=ForwardingSetSelect\left(G(L\left(R_{i}\right),\Phi^{*}\right)$   7:        **for** j=1 to *r* **do**  8:           Calculate EEPA(F,j)  9:        $j_{max}=\underset{j}{\arg\max}\;E\;E\;P\;A\left(F,j\right)$      10:        **for all** $j>j_{max}$ **do**  11:           $F\left(R_{i}\right)=F\left(R_{i}\right)-n_{j}$  12:        $P.ForwardingList\leftarrow F(R_{i})$  13:        ForwardP  14:    **else**  15:        DropP   16:    endif  17:endprocedure  


#### 3.5.3. Forwarding Set Selection

The main idea here is to take advantage of a group of relay nodes, which can simultaneously maximise packet advancement and packet delivery probability. First, we define the expected packet advancement (EPA) to estimate the advancement of each packet that is relayed by a set of nodes. Let $\Phi =\{m_{1},m_{2},...,m_{l}\}$, 1≤l≤c, a subset of nodes with no hidden node, which are decreasingly ordered based on the value of *β* presented in Equation (8). We propose EPA for the subset *Φ*, created by forwarding node $R_{i}$ as follows:

(9) $$E\;P\;A\left(\Phi \right)=\sum_{k=1}^{l}\beta_{ik}P_{ik}\prod_{y=0}^{k-1}{\bar{P}}_{iy}$$

where ${\bar{P}}_{iy}=1-P_{iy}$, $P_{i0}=0$ and $\beta_{ik}$ is the normalised fitness factor, which is calculated based on the relative depth difference between the node $R_{i}$ and each candidate node $m_{k}$ using Equation (8). Now, we aim to extract a forwarding set with maximum EPA from the adjacency graph, which excludes any hidden node.

**Figure 3:**
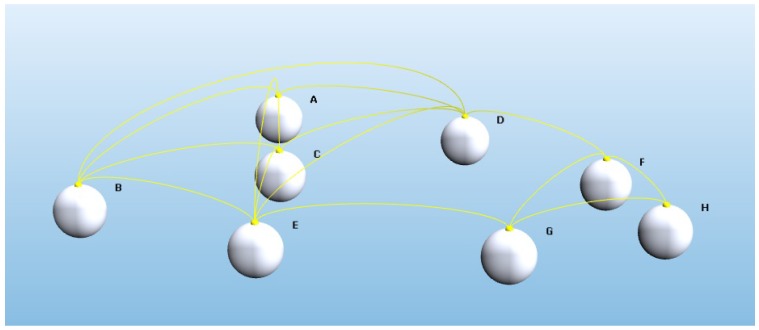
An example of an adjacency graph in a 3D environment.

In the first step of OVAR execution, each forwarding node, $R_{i}$, constitutes its adjacency graph $G(L\left(R_{i}\right))$ with the aid of information provided by beaconing, e.g., the adjacency graph in Figure 3, where the yellow lines show the existence of direct connections between pairs of nodes. In order to remove the possibility of having hidden nodes in a forwarding set, the forwarding node extracts a clique sub-graph from $G(L\left(R_{i}\right))$ with the best EPA to forward the packet. Finding a clique of maximum cardinality in an adjacency graph is an NP-hard problem [40]. Some solutions, exact or heuristic-based, have already been proposed in the literature to address the maximum clique problem. However, every solution comes with some limitations or weaknesses. For instance, an exact solution may deteriorate the performance in order to obtain high accuracy or a heuristic approach may find a local optimum, which is far from a global optimum.

The authors in [41] proposed a greedy randomized adaptive search procedure (GRASP) for the maximum clique problem, which can obtain an acceptable solution in a limited amount of time. Based on this approach, we apply a heuristic that is computationally efficient to extract a clique sub-graph from $G(L\left(R_{i}\right))$ with maximum expected packet advancement. In this way, a greedy randomised search in an iterative manner is performed to find an acceptable solution among the local optimal solutions. The randomisation contributes to generating different solutions in each local search. The greediness of this approach leads to the quick convergence to a local minimum, which decreases the search time in each iteration.

The proposed heuristic includes two phases of construction and local search. Before explaining each phase, it should be noted which criteria are used as a guide for construction. We introduce the probability advance density (PAD) as a metric in each local greedy search, which satisfies all conditions to obtain a better result. Let *C* be a subset of $L(R_{i})$ in which the induced graph G(C) is complete. If $deg_{G(C)}\left(n_{u}\right)$ is the degree of $n_{u}\in C$ with respect to G(C), the PAD of node $n_{u}$ can be calculated as follows:

(10) $$P\;A\;D_{G(C)}\left(n_{u}\right)=P_{iu}\times \beta_{iu}\times deg_{G(C)}\left(n_{u}\right)$$

where $P_{iu}$ and $\beta_{iu}$ are the packet delivery probability and packet advancement between node $R_{i}$ and $n_{u}$, respectively. If a candidate node can maximise this value, this means that it is located in an ideal position in terms of packet delivery and packet advancement and also high connectivity with the rest of the candidate nodes.

In the construction phase, at each step, a node is randomly selected from the candidate nodes with a high PAD value, and other nodes that have no connection to the selected nodes, are removed from the candidate list. Thus, at each step, we will have a clique sub-graph on hand. The construction phase is shown in Algorithm 2. Let *C* be the set of candidate nodes. Initially, all nodes in $L(R_{i})$ are considered as candidates, *i.e.*, $C=L(R_{i})$. There is also a restricted candidate list (RCL), including all candidate nodes with the high PAD value in the sub-graph G(C) induced by the candidate nodes. A node $n_{u}\in C$ is considered to have a high PAD value, if $P\;A\;D_{G(C)}\left(n_{u}\right)$ with respect to G(C) is at least $\delta_{min}+\lambda \left(\delta_{max}-\delta_{min}\right)$, where $\delta_{min}=min\left\{P\;A\;D_{G(C)}\left(n_{u}\right)|n_{u}\in C\right\}$ and $\delta_{max}=max\left\{P\;A\;D_{G(C)}\left(n_{u}\right)|n_{u}\in C\right\}$, and *λ* is a real parameter in the interval [0,1]. Among the candidate nodes in RCL, one node is randomly selected and added to the clique sub-graph under construction. Then, all nodes that are adjacent to the newly selected node will remain in the candidate list, and the rest of the nodes will be removed. This ensures that the new candidate list always includes the nodes that are adjacent to all previously selected nodes in *Φ*. After selecting each node and modifying the candidate list, the PAD value is calculated again for all of the remaining candidate nodes with respect to the new G(C). This procedure will continue until there is no node in the candidate list.

**Algorithm 2** Construction phase.
1:**procedure** Construction($G(L\left(R_{i}\right))$, *λ*, *Φ*)  2:    Setinitialclique *Φ* =∅  3:    Set $C=L(R_{i})$  4:    **while** |C|>0 **do**  5:        G(C)= Sub-graphinducedbyC  6:        $P\;A\;D_{G(C)}\left(n_{u}\right)=P_{iu}\times \beta_{iu}\times deg_{G(C)}\left(n_{u}\right)$   7:        $\delta_{min}=min\left\{P\;A\;D_{G(C)}\left(n_{u}\right)|n_{u}\in C\right\}$  8:        $\delta_{max}=max\left\{P\;A\;D_{G(C)}\left(n_{u}\right)|n_{u}\in C\right\}$  9:        $RCL=\{n_{u}\in C|P\;A\;D_{G(C)}\left(n_{u}\right)\geq \delta_{min}+\lambda \left(\delta_{max}-\delta_{min}\right)\}$  10:        Select $n_{u}$ atrandomfromtheRCL  11:        *Φ* $=\Phi ⋃\{n_{u}\}$  12:        $C=Neighbours_{C}\left(n_{u}\right)$  13:    endwhile   14:endprocedure  


By following a local search after the construction phase, each clique sub-graph is expanded by a simple exchange approach in which a node is removed from the clique sub-graph, *Φ*, if its removal allows two adjacent nodes out of the clique with more contribution in EPA to be included in the clique. Thus, the local search phase can enhance the EPA of each cluster. The details of the local search are described in Algorithm 3. Now, using Algorithm 4, it is shown how these procedures can be used to find a cluster with maximum EPA to forward a packet. In this pseudocode, maxitr indicates the maximum number of iterations. Increasing the number of iterations leads to exploring a better solution. In our problem, note that the adjacency graphs usually include a small number of nodes, and our heuristic approach works very well in small graphs, since iterations can be accomplished more quickly.

The main factor for the complexity of this heuristic is the size of the neighbourhood and the search domain. The complexity of this randomized heuristic depends on the complexity of the heuristic and the number of vertices. Let $n=c=|L(R_{i})|$ denote the number of vertices in the initial adjacency graph. By using a heap to store and update the vertex degrees for sparse graphs, the complexity of this class of heuristic to obtain each independent cluster is given as O(nlogn) [42].

Using this approach, a cluster that can maximise EPA will be selected as the forwarding set ($F(R_{i})$). By utilising this approach, loss probability is decreased, and duplicate transmission paths can disappear efficiently without imposing a high cost to the system.

**Algorithm 3** Local search phase.
1:**procedure** LocalSearch($G(L\left(R_{i}\right))$, *Φ* )  2:    E= Setofedgesin $G(L\left(R_{i}\right))$  3:    $K=\{\left(n_{v},n_{u},n_{w}\right)|n_{v},n_{u},n_{w}\in L\left(R_{i}\right),\left(n_{v},n_{u}\right)\in E,n_{w}\in \Phi ,$ and $n_{v}$ and $n_{u}$             areadjacenttoallnodesin *Φ* except $n_{w},$ $E\;P\;A\left(\Phi ⋃\left\{n_{u},n_{v}\right\}\backslash \left\{n_{w}\right\}\right)>E\;P\;A\left(\Phi \right)\}$  4:    **while** |K|>0 **do**  5:        Select $(n_{v},n_{u},n_{w})\in K$  6:        $\Phi =\Phi ⋃\left\{n_{u},n_{v}\right\}\backslash \left\{n_{w}\right\}$  7:        $K=\{\left(n_{v},n_{u},n_{w}\right)|n_{v},n_{u},n_{w}\in L\left(R_{i}\right),\left(n_{v},n_{u}\right)\in E,n_{w}\in \Phi ,$ and $n_{v}$ and $n_{u}$                  areadjacenttoallnodesinΦ except $n_{w},$ $E\;P\;A\left(\Phi ⋃\left\{n_{u},n_{v}\right\}\backslash \left\{n_{w}\right\}\right)>E\;P\;A\left(\Phi \right)\}$  8:    endwhile   9:endprocedure  


**Algorithm 4** Forwarding set selection.
1:**procedure** ForwardingSetSelect($G\left(L\left(R_{i}\right)\right),\Phi^{*}$)  2:    EPA=−∞  3:    **for** j=1 to maxitr **do**  4:        Select *λ* randomlyfrominterval [0,1]  5:        $Construction(G\left(L\left(R_{i}\right)\right),\lambda ,\Phi )$  6:        $LocalSearch(G\left(L\left(R_{i}\right)\right),\Phi )$   7:        **if** EPA(Φ)>EPA **then**  8:           EPA=EPA(Φ)  9:           $\Phi^{*}=\Phi$  10:        endif   11:    endfor   12:endprocedure  


#### 3.5.4. Reliability and Energy Consumption Trade-Off

Sometimes, especially in a dense network, there are too many nodes in a cluster resulting in wasting of energy. Hence, we introduce a new metric, expected energy and packet advancement (EEPA), to balance energy efficiency and routing efficiency. To consume energy more efficiently, it is assumed that nodes can only listen to a transmitted packet if the packet is destined for them [43]. This is achieved by equipping the nodes with a low power receiver to wake them up to participate in the packet forwarding only by checking the header of the packets. Thus, the receiving energy consumption can be reduced by decreasing the number of receivers in the forwarding set. For instance, if $E_{rx}$ is considered as the receiving energy at each node, by removing *n* nodes from *F*, we can save $n*E_{rx}$ units of energy.

Let EPA(F,j) and E(F,j) be the expected packet advancement and energy consumption of the forwarding set, respectively, when *j* nodes participate in the packet forwarding. The maximum value for EPA and energy ($E\;P\;A_{max}$ and $E_{max}$, respectively) can be obtained by involving all of the nodes in the forwarding set, *i.e.*, EPA(F,r) and E(F,r), where r=∣F∣. In this way, by selecting *j* forwarding candidates from *F*, EEPA can be defined as follows:

(11) $$E\;E\;P\;A\left(F,j\right)=\mu \frac{E\;P\;A(F,j)}{E\;P\;A_{max}}-\rho \frac{E(F,j)}{E_{max}}$$

where *μ* and *ρ* are defined as the weighting coefficients for EPA and energy, respectively. These coefficients can be set according to the desired criteria and density of the network. For instance, if the network is more interested in the energy saving rather than packet delivery, it can increase the *ρ* against the *μ*, or *vice versa*.

The forwarding set should be checked for different numbers of members to achieve the maximum possible value for EEPA. This can be done by examining EEPA for j=1,...,r and, finally, picking the set with the largest value and, accordingly, removing other extra nodes from the forwarding set, if required. In this way, we start from an empty set and add nodes (ordered by their advancement) to the forwarding set one by one. Eventually, the optimal set is selected to relay the packet. In a sparse network, all nodes are held in the forwarding set to increase the reliability; however, in a dense network, some nodes are removed to control the energy dissipation.

#### 3.5.5. Holding Time of Forwarded Packets

Eventually, node $R_{i}$ locally selects the forwarding set $F(R_{i})$ based on our criteria and broadcasts the packet. Algorithm 5 details the receiving packet procedure at each candidate node. OVAR is a sender-based protocol in which the forwarding node decides which candidate nodes should take part in the packet forwarding. The packet header contains all IDs of members of $F(R_{i})$. The receiver node should be in the forwarding set of the sender to accept the packet; otherwise, it drops the packet. Upon receiving a packet by a forwarding candidate, it sets a forwarding timer proportional to its fitness factor (Equation (7)). Because we adopt the retransmission procedure in OVAR, if a node receives a repetitive packet, it should again set a new holding timer for this packet to be synchronised with other candidate nodes in the forwarding set. A node with the highest priority has the lowest forwarding timer value among forwarding candidates, and if the packet is relayed by this node, other lower priorities candidates should discard the packet after hearing the packet transmission. A low priority candidate can become a forwarding node if all of the nodes in the forwarding set with higher priority failed to receive or relay the packet, which can be recognised by listening to the channel. This procedure with the aid of timer scheduling is repeated until the packet is successfully relayed to the next hop. By using this mechanism, redundant transmissions are prevented, which leads to more energy savings for the whole network.

**Algorithm 5** Receiving packet.
1:**procedure** ReceivePacket($R_{i}$, packet)  2:    **if** *$R_{i}.I\;D\in$ header (packet)* **then**  3:        $\text{Calculate}\;\alpha \mathrm{and}T_{hold}$  4:        Setforwardingtimer  5:    **else**  6:        Droppacket   7:    endif  8:endprocedure  


Each candidate node calculates its forwarding timer, $T_{hold}$, as follows:

(12) $$T_{hold}=\frac{1}{2}\left(1-\alpha \right)T_{Delay}+\frac{R-|\vec{SC}|}{\nu_{sound}}$$

where $T_{Delay}$ is the predefined maximum delay, which should be set in a way that all forwarding candidates are able to hear the transmission of higher priority nodes before relaying the packet. *R* and $\nu_{sound}$ are the transmission range of the node and the propagation speed of sound in the water, respectively. $\left|\vec{SC}\right|$ indicates the relative distance between the sending node *S* to the candidate node *C*, which can be estimated based on the received signal strength or time of arrival. The first part of the equation ensures that candidate nodes hold the packet based on their priorities (the greater the fitness factor value, the shorter the timer), and the second part of the equation is used to compensate the receiving delays resulting from the propagation delay between a forwarding node and its multiple candidate nodes. To satisfy the prioritization among candidate nodes after receiving the packet, $T_{Delay}$ in Equation (12) should be big enough to compensate the propagation delay among the forwarding nodes with maximum *R* distance (all candidate nodes are within the transmission range of each other). The prioritization can be guaranteed if $T_{Delay}$ is considered more than $\frac{2R}{\nu_{sound}}$, which is equal to twice as much as the maximum of the propagation delay between the nodes within a forwarding set.

## 4. Energy-Reliability Analysis

**Figure 4:**
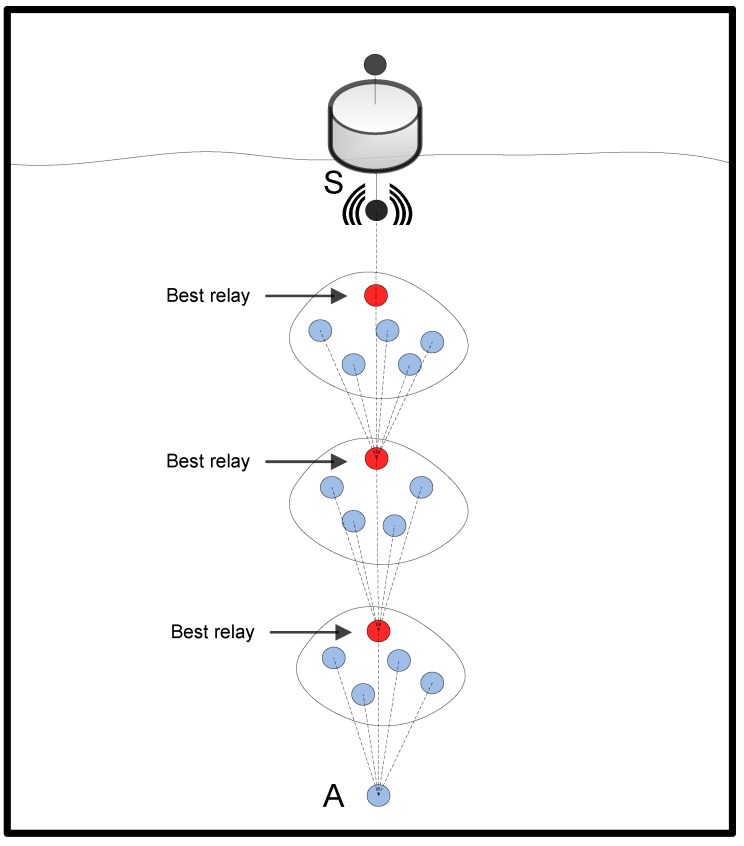
Opportunistic routing in opportunistic void avoidance routing (OVAR).

In this section, we evaluate the optimal trade-off between two objectives: energy consumption and reliability, with regard to the opportunistic data forwarding in OVAR. The analysis carried out indicates that adjusting the number of nodes involved in the data forwarding has a significant impact on the energy consumption and reliability. Without loss of generality, we aim to analyse an opportunistic data forwarding similar to the scenario presented in Figure 4.

As can be seen, a packet is relayed with candidate node collaboration in each cluster. It is assumed that initially, the number of nodes in each cluster is *r*. Let $E_{tx}$ and $E_{rx}$ denote the energy consumed by a node to transmit and receive a packet, respectively. The total amount of energy consumed at the forwarding set *F* with *r* nodes to transmit one packet is given by [44]:

(13) $$E\left(F,r\right)=E_{tx}+r.E_{rx}$$

By removing (r−j) nodes from the cluster, the total amount of energy consumed by the new forwarding set is calculated as follows:

(14) $$E\left(F,j\right)=E_{tx}+j.E_{rx}+\left(r-j\right)E_{re}$$

where $E_{re}$ is the energy used to read the header of the packet for early rejection. By subtracting the E(F,j) from the E(F,r), the amount of energy savings by removing (r−j) nodes is expressed as:

(15) $$ES\left(F,\left(r-j\right)\right)=\left(r-j\right)\left(E_{rx}-E_{re}\right)$$

Consider a network (e.g., Figure 4) with a single-direction progressive routing protocol when the distance between the source and sink is *N* hops. By removing $\nu_{avg}$ nodes from each cluster at each hop, the total savings of energy, $ES_{total}$, for *K* packets can be expressed as:

(16) $$ES_{total}=K.N.\nu_{avg}\left(E_{rx}-E_{re}\right)$$

The above equation shows the total energy savings when no retransmission mechanism is adopted in the forwarding nodes.

If a retransmission procedure is applied, the amount of energy savings depends on two factors: the amount of energy savings by decreasing the number of receiving nodes and the amount of energy loss due to retransmissions. Note that by decreasing the number of receiving nodes at a cluster, the number of retransmissions may be increased due to the increasing of the packet failure probability [45]. Thus, the transmission errors play an important role in reliability and energy savings. A transmission is successful if at least one node in each cluster can receive the packet without any error. Instead of using an acknowledgement mechanism, which is costly in UWSNs, underwater nodes can easily notice a successful delivery just by listening to the neighbouring nodes’ transmissions. For packet transmission suppression, candidate nodes only read the header of the packet.

**Figure 5:**
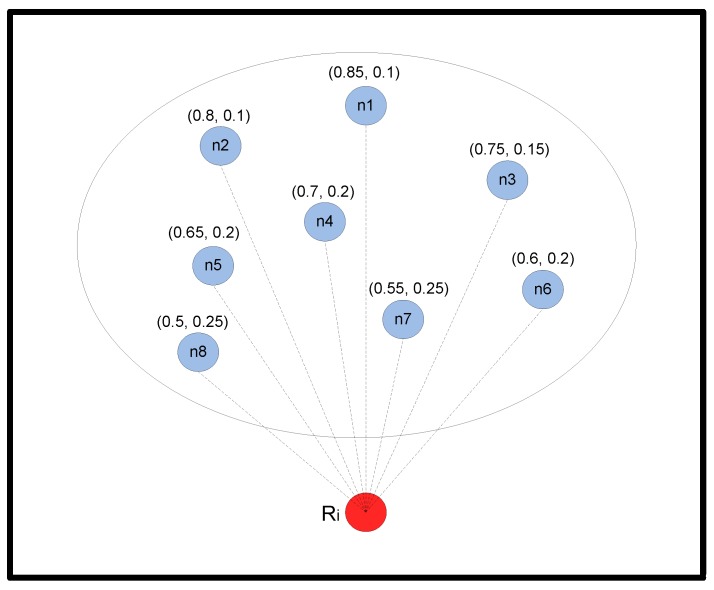
Example of opportunistic data forwarding in one hop.

**Figure 6:**
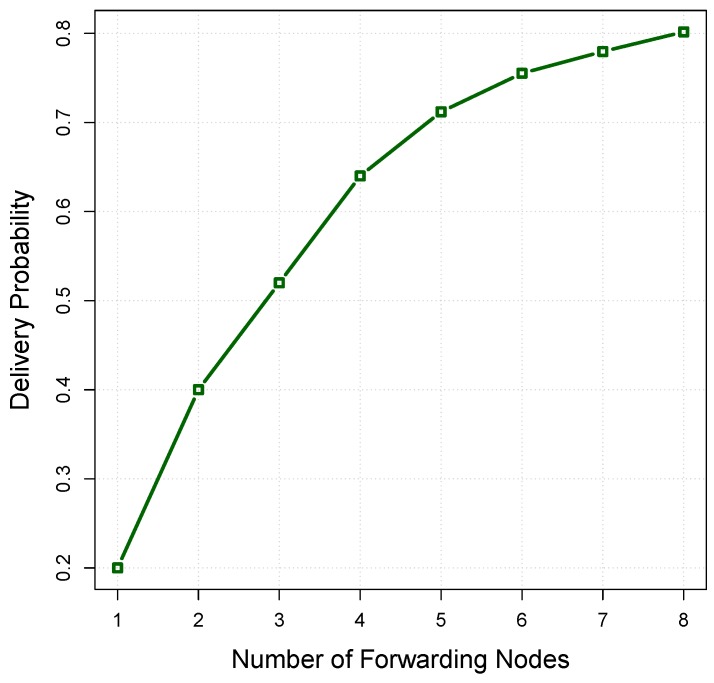
The relationship between the number of forwarding nodes and the delivery probability.

If a packet is relayed with the collaboration of *l* nodes in the forwarding set, the successful transmission probability can be calculated as follows [45]:

(17) $$P_{s}=\sum_{i=1}^{l}P_{i}\prod_{j=1}^{i-1}\left(1-P_{i}\right)$$

where $P_{i}$ is the packet delivery probability between the forwarding node and receiving node. Note that the forwarding priority of candidate nodes affects the value of $P_{s}$. To clarify the matter, consider the opportunistic data forwarding in Figure 5, as an example. The forwarding set of $R_{i}$ includes a number of candidate nodes for which each of them is associated with a pair, $\left(\beta_{ij},P_{ij}\right)$, where $\beta_{ij}$ is the packet advancement and $P_{ij}$ is the packet delivery probability between $R_{i}$ and $n_{j}$. If candidate nodes are sorted based on the value of $\beta_{ij}.P_{ij}$ and added to the forwarding set, the successful transmission probability is changed in accordance with the diagram shown in Figure 6, which shows the effect of adding every individual node into the forwarding set on the probability of successful packet delivery.

**Figure 7:**
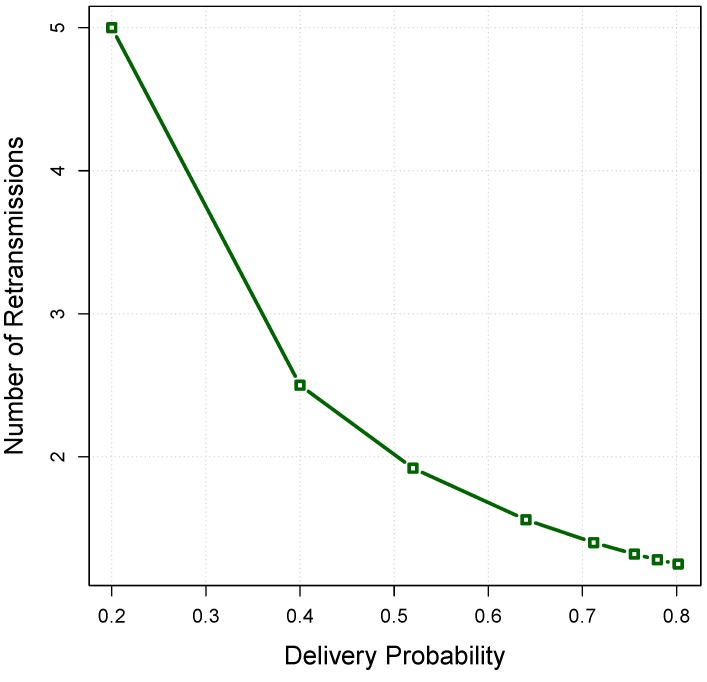
The relationship between the delivery probability and the number of retransmissions.

**Figure 8:**
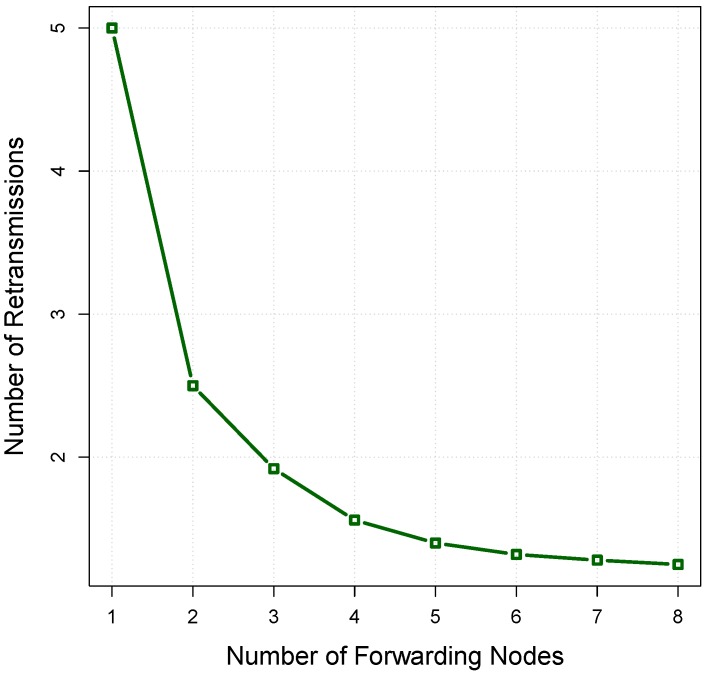
The relationship between the number of forwarding nodes and the number of retransmissions.

If there is no limitation on the number of retransmissions to deliver a packet, the average transmissions number, $\bar{X}$, is equal to the sum of a geometric series expressed as follows [45]:

(18) $$\bar{X}=\sum_{n=1}^{\infty }n.P_{s}.{\left(1-P_{s}\right)}^{\left(n-1\right)}=\frac{1}{P_{s}}$$

where $P_{s}$ is the successful transmission probability of the data packet and *n* is the number of transmissions. At each hop, the expected number of transmissions is $\bar{X}$; if the transmitter-receiver distance *d* is selected at each hop, the expected number of transmissions from the source to the sink node, $\bar{T\;X}$, is given by:

(19) $$\bar{T\;X}=\bar{X}\frac{d_{src-sink}}{d}$$

where $d_{src-sink}$ is the distance between the source and sink. Figure 7 shows the relationship between the number of retransmissions and the delivery probability obtained from Figure 6. In this model, the increased number of retransmissions by removing (r−j) nodes from the forwarding set *F* can be estimated as follows:

(20) $$▵X=\frac{1}{P_{j}}-\frac{1}{P_{r}}$$

where $P_{r}$ and $P_{j}$ are the packet delivery probability with *r* nodes and *j* nodes in the forwarding set, respectively. Based on the derived relations between the Figure 6 and Figure 7, the relationship between the number of forwarding nodes and the number of retransmissions is shown in Figure 8. In an opportunistic data forwarding equipped with a retransmission mechanism, the total amount of energy consumed at cluster *Φ* with *l* nodes to transmit one packet, $E^{ret}$, is expressed as follows:

(21) $$E^{ret}\left(\Phi ,l\right)=\bar{X}\left(E_{tx}+l.E_{rx}\right)$$

**Figure 9:**
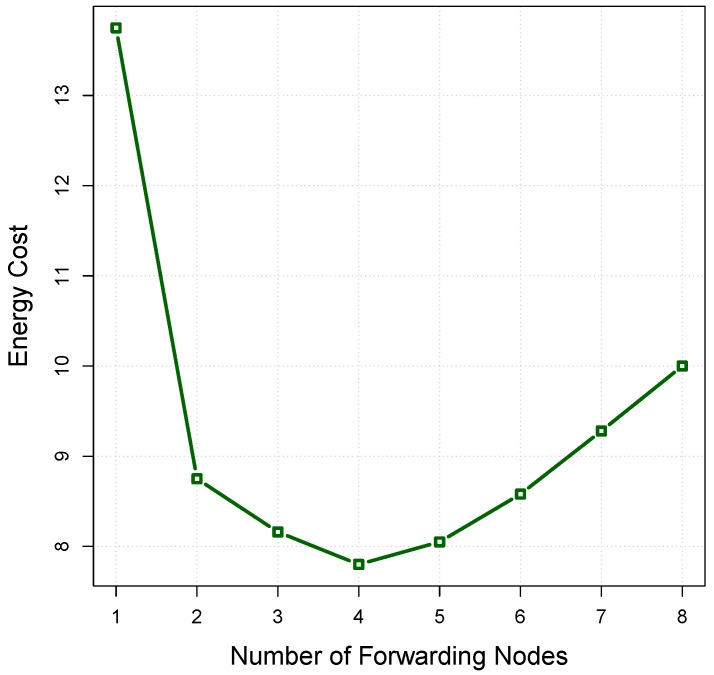
The relationship between the number of forwarding nodes and energy cost.

The relation between the energy consumption and the number of forwarding nodes is shown in Figure 9 by assuming $E_{tx}=2$ units, $E_{rx}=0.75$ units. It is obvious that the number of forwarding nodes has a vital impact on the energy cost of the set. Lack of sufficient forwarding nodes may increase the number of retransmissions and, subsequently, the energy cost. On the other hand, an excessive number of forwarding nodes can also waste energy. In this example, the energy cost reaches its minimum level, at l=4, where the corresponding ordered node set is $\langle n_{4},n_{7},n_{5},n_{8}\rangle$, which among them, $n_{4}$ and $n_{8}$ have the highest and lowest relay priority, respectively.

Assuming that by removing $\nu_{avg}$ nodes from each cluster, the number of retransmissions is increased by $\theta_{avg}$, then the amount of energy loss at each hop, on average, can be estimated as follows:

(22) $$▵E_{rt}=\theta_{avg}.\left(E_{tx}+\left(r_{avg}-\nu_{avg}\right).E_{rx}\right)$$

where $r_{avg}$ indicates the maximum number of nodes, on average, in the initial forwarding set. Note that $\theta_{avg}$ is calculated using Equation (20). If a retransmission procedure is adopted, the total energy savings in Equation (16) turns into the following equation:

(23) $$ES_{total}^{ret}=K.N.\left(\nu_{avg}\left(E_{rx}-E_{re}\right)-▵E_{rt}\right)$$

In this model, the energy savings is confined by the energy loss of the retransmissions. By removing too many nodes from each forwarding set, the packet failure probability increases, and subsequently, more energy will be required to maintain the reliability. On the other hand, if the majority of nodes are kept in the forwarding set, the reliability is enhanced, but a significant amount of energy is wasted because of involving many receiving nodes. Therefore, the number of candidate nodes within a forwarding set should be selected carefully with respect to the energy and reliability constraints. The OVAR protocol is able to satisfy these constraints by using the heuristic approach proposed in Section 3.5.4.

## 5. Experimental Results

The details of our simulation study and also the performance results are presented in this section. We use Aqua-Sim [46], an NS-2-based simulating software for underwater acoustic networks, to evaluate OVAR against VBF, HHVBF and VAPR in a single-sink architecture.

### 5.1. Simulation Setup

The performance of routing protocols somehow depends on the underlying MAC protocol. Similar to the majority of studies in this field, we use the Carrier Sense Multiple Access (CSMA) MAC protocol without using its RTS/CTS (Request to Send/Clear to Send) and ACK mechanism. In this way, a forwarding node can broadcast a packet if the channel is free; otherwise, it will back off. The packet will be discarded if the forwarding node backs off five times. For suppressing the redundant transmissions, nodes can simply listen to the channel and drop the packet if it is relayed by other candidates.

In our simulations, we consider the channel model described in Section 3.2 to simulate a lossy underwater environment. The transmission power is set to 95 dB re *μ* Pa, and the transmission range for all nodes is considered as 100 m. The data generation rate is set to one packet per second, which can effectively prevent the interference of two continuous packets. The channel bit rate is 10 kbps, and the propagation speed of the acoustic signal in the underwater environment is 1500 m/s. The size of packets varies by changing the number of forwarding candidates, but its average value is less than 150 B. The coefficients of EPA and energy (*μ*,*ρ*) in Equation (11) are considered equal to balance energy and routing efficiency. We set $T_{Delay}$ in Equation (12) as one second based on our model. In the beaconing procedure, $T_{update}$ is set to 30 seconds, and $T_{invalid}$ is considered as 75 seconds (considering the random jitters to prevent synchronization).

**Figure 10:**
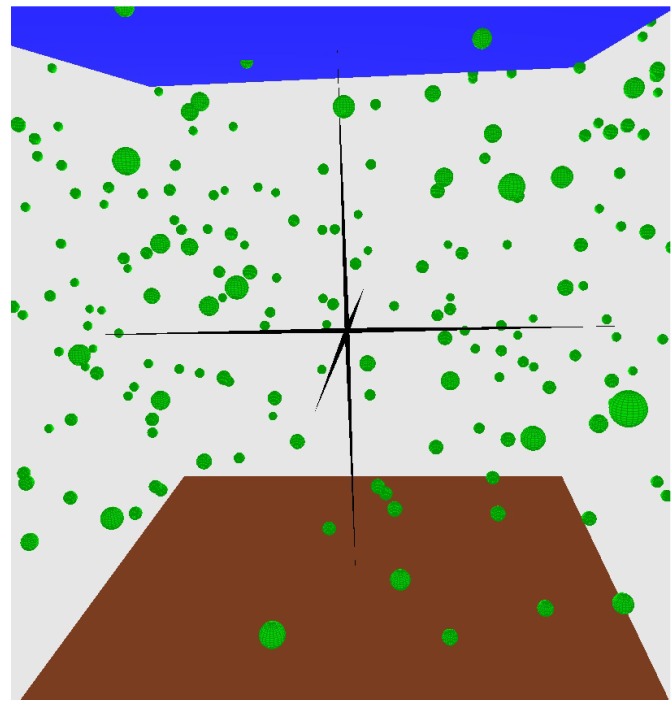
Underwater 3D environment.

The relay nodes (ranging from 400 to 1200) are randomly deployed in a 500 m × 500 m × 1000 m 3D field. Relay nodes can move horizontally at the speed of 2 m/s by following a random walk 2D mobility model (moving in the X-Y plane), which is mostly used by the underwater routing protocols (e.g., VBF, HHVBF, AHH-VBF, DBR) [22,26,27,28]. Furthermore, we consider a single sink at location (100; 100; 0) to collect the information and a source node at location (400; 400; 1000) to generate the packets to be transferred to the sink node. The sink and source are intentionally placed at opposite corners of the field to have better assessment of the routing protocols. We consider the maximum pipeline radius for VBF and HHVBF as 100 m (equal to the transmission range of nodes) in which they have the highest performance in packet delivery. All of the results are averaged over 20 runs for randomly-generated topologies with the 95% confidence interval. The simulation time for each run is set to 1000 s. An example of a network topology is presented in Figure 10.

### 5.2. Results and Analysis

In this section, we evaluate the performance of OVAR against those of VBF, HHVBF and VAPR in terms of packet delivery ratio, energy tax, end-to-end delay, average hop count and propagation deviation factor.

**Figure 11:**
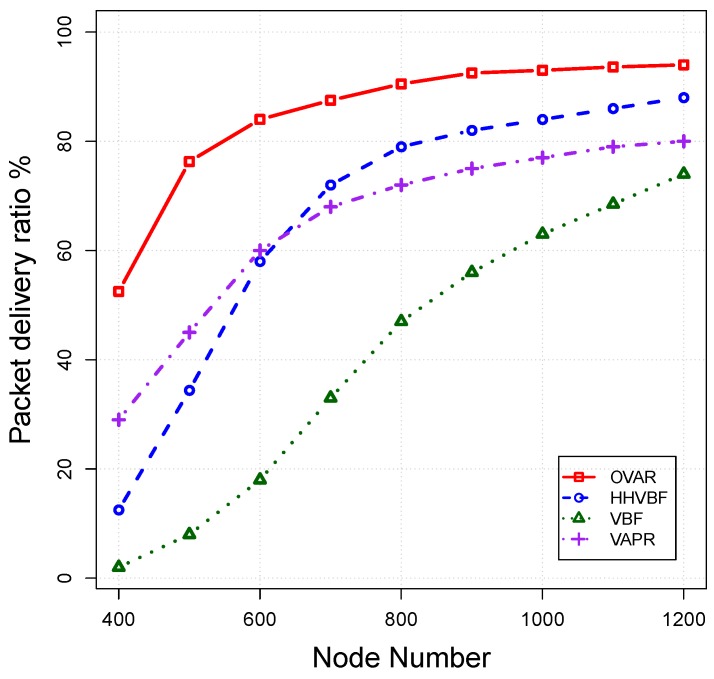
Packet delivery ratio *vs*. node density.

*Packet delivery ratio (PDR):* This is defined as the ratio of the number of packets successfully received by the sink node to the number of packets generated by the source. The results for the packet delivery ratio at different node densities are shown in Figure 11. PDR is increased by increasing the number of nodes, because it reduces the size of void areas and also their number, In a dense network, more forwarding nodes have this chance to be placed in the routing path, and consequently, the PDRs of routing protocols converge to a high value. On the contrary, the majority of nodes in sparse networks are disconnected, which leads to lower PDR. OVAR always has higher PDR than that of other routing protocols (especially in a sparse network), because it inherently excludes all of the routes leading to a void area and enhances the packet delivery probability in each step towards the destination. However, in VBF and HHVBF protocols, packet failure is increased when the void area appears in their routing pipes. Furthermore, these protocols do not take into account the packet delivery probability as a criteria for forwarding nodes’ selection. In VAPR, when the network is sparse, it has a better packet delivery ratio in comparison to that of HHVBF, but it falls below HHVBF by increasing the network density. It should be mentioned that VAPR is an efficient approach in a multi-sink architecture, while its performance is diminished in a single-sink architecture.

**Figure 12:**
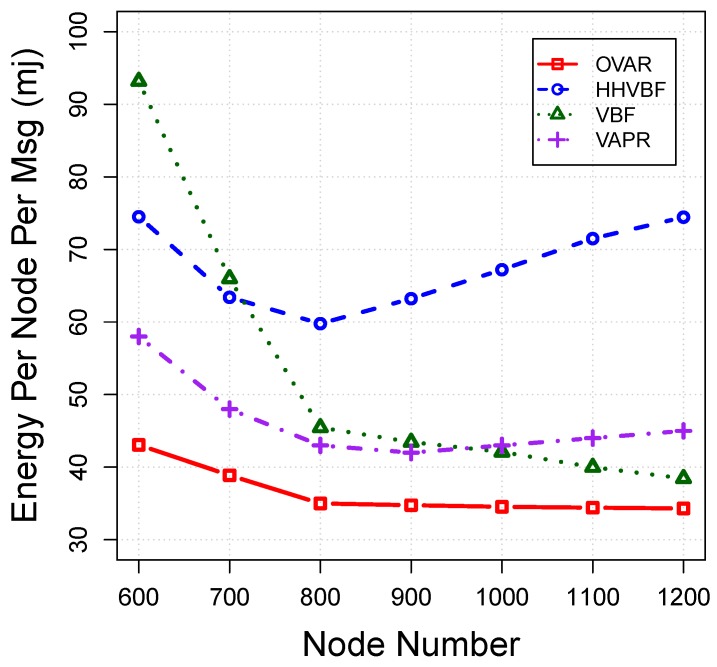
Energy consumption per message *vs*. node density.

*Energy tax:* We measure the energy tax in millijoules (mj) in terms of energy spent per node and per message to route a packet towards the destination (including energy for sending mode, receiving mode and idle mode). Figure 12 plots the energy tax for each protocol *versus* the number of nodes. As can be seen, OVAR consumes lower energy than other protocols for the delivery of each packet to the sink. This is due to the fact that OVAR confines the forwarding candidates in a cluster (without hidden nodes), which can prevent redundant packet transmissions and collisions. However, the radius of the pipe in VBF and HHVBF has a great impact on the total energy consumption and packet delivery ratio. Selecting a large radius can involve more nodes in packet forwarding; however, it increases duplicated packets, which leads to more energy waste. On the other hand, a lower radius causes more packet failures. In contrast to our approach, using pipelines for opportunistic routing is not able to achieve an appropriate trade-off between lower energy consumption and a higher packet delivery ratio. In sparse networks, the energy tax of VBF and HHVBF is high due to the low packet delivery ratio of them. Although VAPR does not have the duplicated packets problem, it still consumes more energy than OVAR. This is due to the fact that the number of forwarding nodes in the forwarding set is not adjusted according to the network density. VAPR also should periodically measure all distances to its neighbouring nodes to be used for forwarding set selection and forwarding timers’ calculation, resulting in more energy consumption. In terms of normalised energy consumption, OVAR is highly efficient and achieves a high delivery ratio over its consumed energy. In dense networks (while the delivery ratio has almost reached the maximum), increasing the number of nodes has little contribution to the packet delivery ratio, but wastes energy. However, OVAR mitigates this energy waste by using a principled approach to modify the forwarding set. To achieve this, in dense networks, the number of forwarding nodes is slightly increased or held constant by OVAR, to control the energy dissipation.

**Figure 13:**
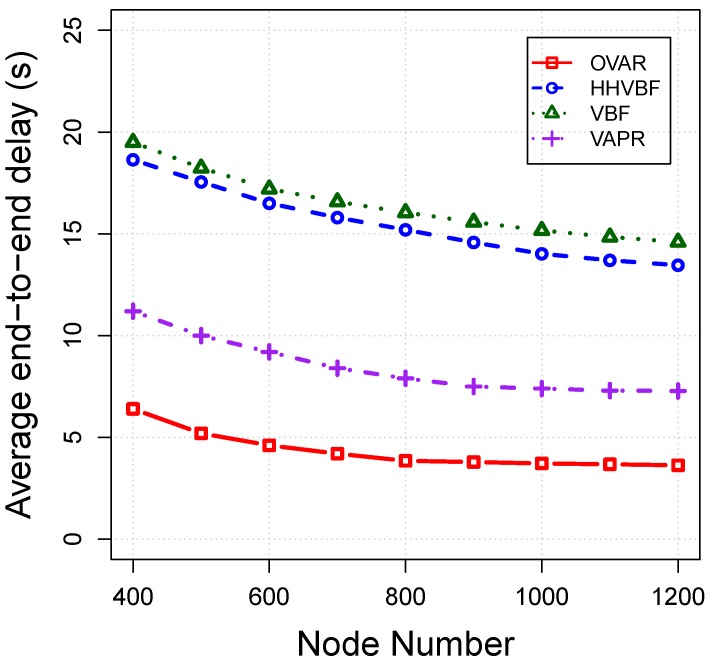
Average end-to-end delay *vs*. node density.

*Average end-to-end delay:* This criteria measures the average delay time taken from the moment of the creation of packets at the source until being received by the sink for all of the successfully-received packets. We take into account the propagation delay, transmission delay and holding time of packets for calculating the end-to-end delay. The average end-to-end delay for each protocol is plotted in Figure 13. The average end-to-end delay for all protocols decreases by increasing the number of nodes, because the forwarding node can find more qualified nodes in its neighbourhood. The latency of OVAR is very small in comparison to other protocols, because packets almost use the optimal path towards the sink with the least possible transmissions. However, in VBF and HHVBF, nodes with better progress towards the sink may be located at the outside of the pipe, and ignoring them can increase the latency. Moreover, VBF and HHVBF only give higher priority to the nodes that are close to the virtual vector (which is drawn from the source or sender to the sink) and not necessarily the nodes with a lower hop count distance to the sink. On the other hand, VAPR imposes more delay on packets in comparison to OVAR, because it is not flexible enough to forward the packet in any direction and only can maximise expected packet advancement upward or downward (according to the selected direction). This feature of VAPR increases its delay despite the use of reachability information. Furthermore, in OVAR, each node can hold a packet with less average holding time (by setting a lesser amount of $T_{Delay}$) due to the fact that candidate nodes are closer to each other on average. However, the desirableness factor (a predefined maximum delay) of VBF and HHVBF is obviously longer than that of our method because of the different ways of the selection and prioritization of the forwarding nodes. VAPR also takes advantage of the ACK mechanism, which increases its delay. In OVAR, the number of collisions and retransmissions reach the least amount possible, and this improves the packet delivery time. However, in VBF and HHVBF, forwarding candidates may be located at different sides of the pipe, and because of the hidden nodes, collisions will be increased at the receiver. As a result, only the packets that avoid the collisions (by using the back-off process) can successfully be delivered to the sink. Thus, the latency of VBF and HHVBF is increased by increasing the number of retransmissions due to the existence of hidden nodes in the pipe. On the other hand, the VAPR approach is not reliable enough to completely remove all of the hidden nodes from the forwarding set. This is due to the fact that VAPR constructs the forwarding set based on the estimated distances of two hops’ connectivities, which is not sufficiently precise in comparison to the OVAR approach.

**Figure 14:**
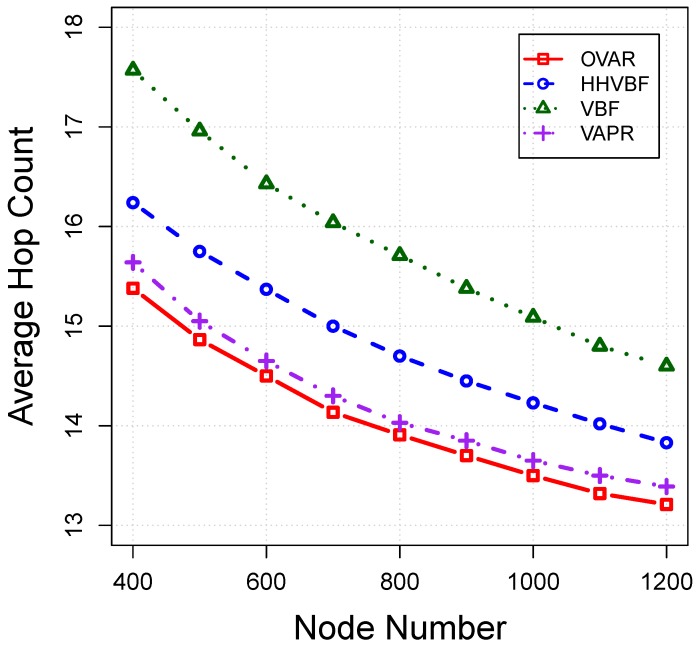
Average hop count *vs*. node density.

*Average hop count:* This shows the average number of hops on the routing path from the source to the sink. Involving as few as possible nodes to deliver a packet is a desirable factor. An efficient routing protocol should find its path toward the destination with the least possible number of relay nodes (e.g., minimum number of transmissions). Ideally, a packet should not be directed toward a void area. However, this is not always possible for some algorithms, like VBF and HHVBF. Figure 14 shows that the number of hops to reach the destination is varied with changing the node density. In sparse scenarios, the shortest line between the source and sink is not always covered by some nodes, and consequently, a longer path, with more number of hops, should be taken to reach the destination. However, as the network becomes denser, a lesser number of hops is on average required; the chance of finding the routes with the least number of hops is also higher. As can be seen, OVAR can deliver packets to the destination by involving lower intermediate nodes due to its global view of the network topology and its flexibility to change the forwarding direction. However, the VBF and HHVBF routing protocols are confined to their pipelines and are not flexible enough to find a path with a minimal number of hops to the sink. Furthermore, the discovered path by VAPR has more hop counts compared to OVAR, because it is only performed based on the directional trails and not on the basis of the hop count value.

**Figure 15:**
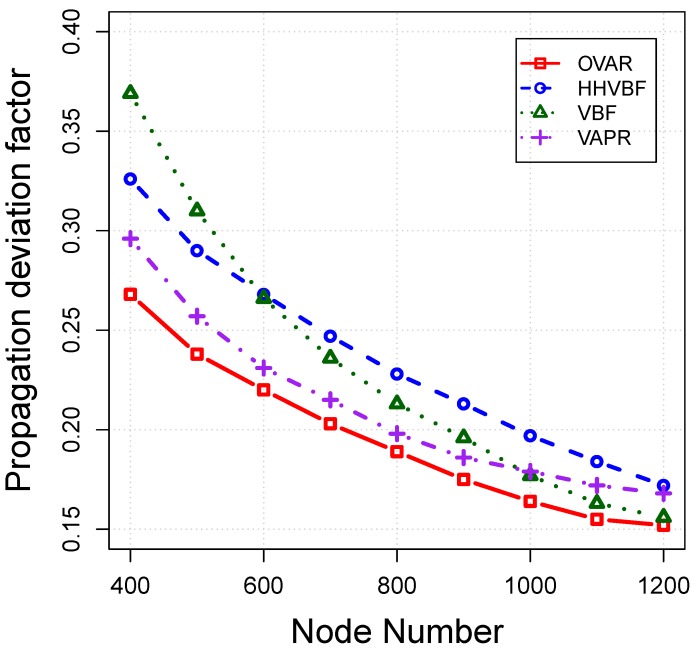
Propagation deviation factor *vs*. node density.

*Propagation deviation factor:* This is a normalized value for showing the total traversed distance by each packet from the source to the sink and can be calculated using [22]:

(24) $$Pdf=\frac{TD-SD}{SD}$$

where TD and SD are total travelled distance and the straight line distance from the source to the sink node, respectively. The propagation deviation factor is increased if packets are delivered to the destination through a longer distance. An efficient routing protocol selects the best path, or one very close to the best one, with the lowest propagation deviation value out of many available routes to the destination. The propagation deviation factor of routing protocols is shown in Figure 15. By increasing the number of nodes, the shorter routing paths can be established by the routing protocols. As can be seen, OVAR has obtained the lowest value in comparison to other routing protocols. This indicates that hop count and depth are adequately combined to reduce the traversed distance. The advantage of our method is that it can reduce the traversed distance without any knowledge about the node coordinates. On the other hand, the paths discovered by VBF and HHVBF are far away from the optimal path in sparse networks. Nonetheless, VBF can obtain a good propagation deviation factor in a dense network when its constant pipeline includes the sufficient number of nodes close to the straight line between the source and sink. Although VAPR avoids all kinds of voids in its routing path, it is not always interested in the forwarding nodes closer to the sink (direct line between the forwarding node and sink), but the nodes with more advancement toward up or down, depending on the directional trails.

**Figure 16:**
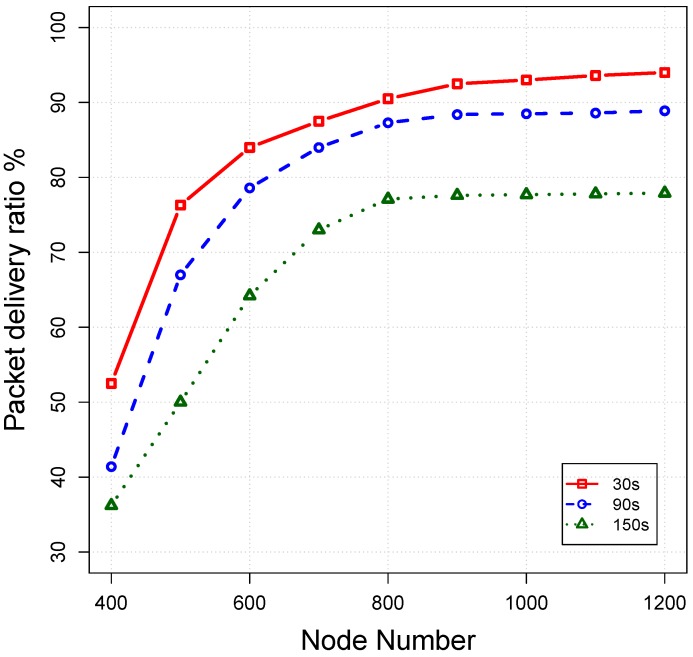
Impact of different beacon intervals on the packet delivery ratio.

**Figure 17:**
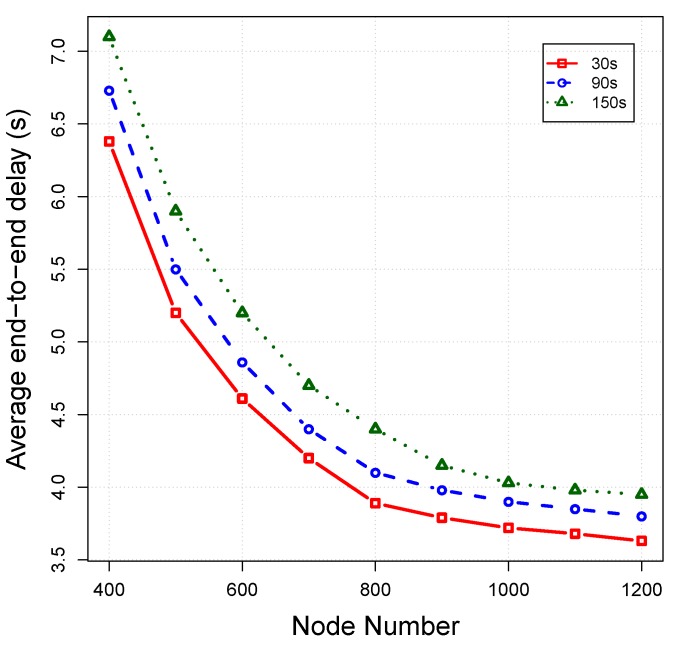
Impact of different beacon intervals on the average end-to-end delay.

**Figure 18:**
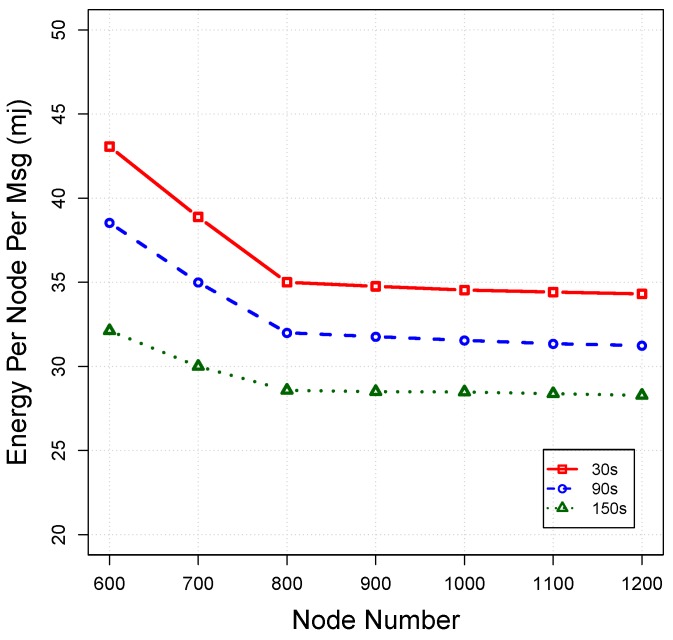
Impact of different beacon intervals on the energy consumption.

*Impact of beaconing interval:* In order to evaluate the impact of beacon intervals on OVAR performance, we conduct extensive simulations at varied beacon intervals of 30 s, 90 s and 150 s under the same operational condition as before. The impacts of different beacon intervals on the packet delivery ratio, average end-to-end delay and energy consumption are shown in Figure 16, Figure 17 and Figure 18, respectively.

As can be seen in Figure 16, by increasing the beacon interval, the packet delivery ratio is decreased because the routing and neighbourhood information gradually become outdated with the passing of time. Furthermore, by considering the longer intervals, all, of the estimates about the packet delivery probabilities can become obsolete due to node movement. Thus, the beacon interval should be set in a way that the packet delivery ratio reaches the highest possible value without imposing high overhead to the network. On the other hand, late updating can potentially increase the latency of received packets. This is due to the fact that packets are relayed over the non-optimal paths because the forwarding decisions are partially based on the outdated information. As depicted in Figure 18, energy consumption is further decreased by postponing the beaconing at the cost of deteriorating other performance metrics.

## 6. Conclusions

Addressing the void issue in 3D acoustic UWSNs is a challenging task when designing routing protocols. In this paper, it has been shown that including a preventative void-handling technique by utilising soft-state information can significantly increase the routing protocols’ performance. We have also investigated opportunistic routing to show how it can overcome the drawback of unreliable acoustic transmission by taking advantage of intermediate nodes’ collaboration to relay packets. To this end, we have proposed OVAR, an opportunistic routing protocol, to minimise the number of dropped packets by efficiently bypassing void areas and also to maximise the transmission reliability where there exists significant ambient noises and channel fading. OVAR exploits the local information obtained from the periodic beaconing, constructs the adjacency graph, selects the best forwarding set by removing the possibility of having hidden nodes in the cluster after applying a low-cost heuristic solution and, finally, adjusts the number of forwarding nodes within the cluster based on the energy-reliability trade-off constraints. Finally, different timer values, proportional to the depth difference of members, are assigned to each member of the forwarding set to specify the priority of each node to relay the packet. In contrast to most of the protocols reported in the field, which route packets only toward the surface, OVAR can route packets in any direction to guarantee smooth bypassing of any type of void areas. Our simulation results have demonstrated that OVAR significantly decreases packet loss, energy consumption, end-to-end delay, hop count and traversed distance in sparse to dense scenarios. As future work, we plan to investigate the relationship between the opportunistic data forwarding and network energy balance based on the residual energy distribution in the entire network.

## References

[1] Heidemann J., Ye W., Wills J., Syed A., Li Y. Research challenges and applications for underwater sensor networking. Proceedings of the IEEE Wireless Communications and Networking Conference (WCNC 2006).

[2] Akyildiz I.F., Pompili D., Melodia T. (2005). Underwater acoustic sensor networks: Research challenges. Ad Hoc Netw..

[3] Akyildiz I.F., Pompili D., Melodia T. (2004). Challenges for efficient communication in underwater acoustic sensor networks. ACM Sigbed Rev..

[4] Vasilescu I., Kotay K., Rus D., Dunbabin M., Corke P. (2005). Data collection, storage, and retrieval with an underwater sensor network. Proceedings of the 3rd International Conference on Embedded Networked Sensor Systems.

[5] Ayaz M., Baig I., Abdullah A., Faye I. (2011). A survey on routing techniques in underwater wireless sensor networks. J. Netw. Comput. Appl..

[6] Pompili D., Akyildiz I.F. (2009). Overview of networking protocols for underwater wireless communications. IEEE Commun. Mag..

[7] Kheirabadi M.T., Mohamad M.M. (2013). Greedy routing in underwater acoustic sensor networks: A survey. Int. J. Distrib. Sens. Netw..

[8] Noh Y., Lee U., Wang P., Choi B.S.C., Gerla M. (2013). VAPR: Void-aware pressure routing for underwater sensor networks. IEEE Trans. Mob. Comput..

[9] Fang Q., Gao J., Guibas L.J. (2006). Locating and bypassing holes in sensor networks. Mob. Netw. Appl..

[10] Chen D., Varshney P.K. (2007). A survey of void handling techniques for geographic routing in wireless networks. IEEE Commun. Surv. Tutor..

[11] Xie P., Zhou Z., Peng Z., Cui J.H., Shi Z. (2009). Void Avoidance in Three-Dimensional Mobile Underwater Sensor Networks. Wireless Algorithms, Systems, and Applications.

[12] Coutinho R.W., Boukerche A., Vieira L.F., Loureiro A.A. (2015). A novel void node recovery paradigm for long-term underwater sensor networks. Ad Hoc Netw..

[13] Stojanovic M. (2007). On the relationship between capacity and distance in an underwater acoustic communication channel. ACM SIGMOBILE Mob. Comput. Commun. Rev..

[14] Lee U., Wang P., Noh Y., Vieira F., Gerla M., Cui J.H. Pressure routing for underwater sensor networks. Proceedings of the IEEE INFOCOM.

[15] Zorzi M., Casari P., Baldo N., Harris A.F. (2008). Energy-efficient routing schemes for underwater acoustic networks. IEEE J. Sel. Areas Commun..

[16] Li Z., Yao N., Gao Q. (2014). Relative Distance Based Forwarding Protocol for Underwater Wireless Networks. Int. J. Distrib. Sens. Netw..

[17] Lloret J. (2013). Underwater sensor nodes and networks. Sensors.

[18] Liu H., Zhang B., Mouftah H.T., Shen X., Ma J. (2009). Opportunistic routing for wireless ad hoc and sensor networks: Present and future directions. IEEE Commun. Mag..

[19] Vieira L.F.M. Performance and trade-offs of opportunistic routing in underwater networks. Proceedings of the IEEE Wireless Communications and Networking Conference (WCNC 2012).

[20] Patel T., Kamboj P. Opportunistic routing in wireless sensor networks: A review. Proceedings of the IEEE Advance Computing Conference (IACC 2015).

[21] Nguyen S.T., Cayirci E., Yan L., Rong C. (2009). A shadow zone aware routing protocol for acoustic underwater sensor networks. IEEE Commun. Lett..

[22] Yu H., Yao N., Liu J. (2014). An adaptive routing protocol in underwater sparse acoustic sensor networks. Ad Hoc Netw..

[23] Chandrasekhar V., Seah W.K., Choo Y.S., Ee H.V. (2006). Localization in underwater sensor networks: Survey and challenges. Proceedings of the 1st ACM International Workshop on Underwater Networks.

[24] Han G., Jiang J., Shu L., Xu Y., Wang F. (2012). Localization algorithms of underwater wireless sensor networks: A survey. Sensors.

[25] Zhou Z., Cui J.H., Zhou S. (2007). Localization for Large-Scale Underwater Sensor Networks. Networking 2007. Ad Hoc and Sensor Networks, Wireless Networks, Next Generation Internet.

[26] Yan H., Shi Z.J., Cui J.H. (2008). DBR: Depth-Based Routing for Underwater Sensor Networks. NETWORKING Ad Hoc and Sensor Networks, Wireless Networks, Next Generation Internet.

[27] Xie P., Cui J.H., Lao L. (2006). VBF: Vector-Based Forwarding Protocol for Underwater Sensor Networks. Networking 2006. Networking Technologies, Services, and Protocols; Performance of Computer and Communication Networks; Mobile and Wireless Communications Systems.

[28] Nicolaou N., See A., Xie P., Cui J.H., Maggiorini D. Improving the robustness of location-based routing for underwater sensor networks. Proceedings of the IEEE OCEANS Europe.

[29] Darehshoorzadeh A., Boukerche A. (2015). Underwater sensor networks: A new challenge for opportunistic routing protocols. IEEE Commun. Mag..

[30] Coutinho R.W., Boukerche A., Vieira L.F., Loureiro A. GEDAR: Geographic and opportunistic routing protocol with depth adjustment for mobile underwater sensor networks. Proceedings of the IEEE International Conference on Communications (ICC 2014).

[31] Coutinho R.W., Vieira L.F., Loureiro A. DCR: Depth-Controlled routing protocol for underwater sensor networks. Proceedings of the IEEE Computers and Communications (ISCC 2013).

[32] Guangzhong L., Zhibin L. Depth-based multi-hop routing protocol for underwater sensor network. Proceedings of the IEEE 2nd International Conference on Industrial Mechatronics and Automation (ICIMA 2010).

[33] Wahid A., Kim D. (2012). An energy efficient localization-free routing protocol for underwater wireless sensor networks. Int. J. Distrib. Sens. Netw..

[34] Gao M., Foh C.H., Cai J. (2012). On the selection of transmission range in underwater acoustic sensor networks. Sensors.

[35] Domingo M.C., Prior R. (2008). Energy analysis of routing protocols for underwater wireless sensor networks. Comput. Commun..

[36] Raghunathan V., Schurgers C., Park S., Srivastava M.B. (2002). Energy-aware wireless microsensor networks. IEEE Signal Process. Mag..

[37] Anastasi G., Conti M., di Francesco M., Passarella A. (2009). Energy conservation in wireless sensor networks: A survey. Ad Hoc Netw..

[38] Rodoplu V., Park M.K. An energy-efficient MAC protocol for underwater wireless acoustic networks. Proceedings of the MTS/IEEE OCEANS.

[39] Chen K., Ma M., Cheng E., Yuan F., Su W. (2014). A survey on MAC protocols for underwater wireless sensor networks. IEEE Commun. Surv. Tutor..

[40] Abello J., Pardalos P., Resende M. (1998). On Maximum Clique Problems in Very Large Graphs.

[41] Feo T.A., Resende M.G. (1995). Greedy randomized adaptive search procedures. J. Glob. Optim..

[42] Feo T.A., Resende M.G., Smith S.H. (1994). A greedy randomized adaptive search procedure for maximum independent set. Oper. Res..

[43] Harris A.F., Stojanovic M., Zorzi M. (2006). When underwater acoustic nodes should sleep with one eye open: Idle-time power management in underwater sensor networks. Proceedings of the 1st ACM International Workshop on Underwater Networks.

[44] Zeng K., Lou W., Yang J., Brown D.R. On geographic collaborative forwarding in wireless ad hoc and sensor networks. Proceedings of the IEEE International Conference on Wireless Algorithms, Systems and Applications (WASA 2007).

[45] Zhang R., Berder O., Sentieys O. (2013). Energy-Latency Tradeoff of Opportunistic Routing. Routing in Opportunistic Networks.

[46] Xie P., Zhou Z., Peng Z., Yan H., Hu T., Cui J.H., Shi Z., Fei Y., Zhou S. Aqua-Sim: An NS-2 based simulator for underwater sensor networks. Proceedings of the IEEE OCEANS 2009, MTS/IEEE Biloxi-Marine Technology for Our Future: Global and Local Challenges.

